# Development of an Efficient Voltammetric Sensor for the Monitoring of 4-Aminophenol Based on Flexible Laser Induced Graphene Electrodes Modified with MWCNT-PANI

**DOI:** 10.3390/s22030833

**Published:** 2022-01-22

**Authors:** Salem Nasraoui, Sami Ameur, Ammar Al-Hamry, Mounir Ben Ali, Olfa Kanoun

**Affiliations:** 1 Professorship of Measurement and Sensor Technology, Chemnitz University of Technology, 09111 Chemnitz, Germany; ammar.al-hamry@etit.tu-chemnitz.de; 2 Centre for Research on Microelectronics and Nanotechnology of Sousse, Tunisia and NANOMISENE Lab, LR16CRMN01, University of Sousse Sahloul, Sousse 4003, Tunisia; sami.ameur@ISSACM.u-sousse.tn (S.A.); mounir.benali@issatso.rnu.tn (M.B.A.); 3 Higher Agronomic Institute of Chott-Mariem, University of Sousse, Sousse 4034, Tunisia; 4 Higher Institute of Applied Sciences and Technology of Sousse, University of Sousse, Sousse 4003, Tunisia

**Keywords:** laser induced carbon, MWCNT-PANI, electrochemical sensor, 4-Aminophenol

## Abstract

Sensitive electrodes are of a great importance for the realization of highly performant electrochemical sensors for field application. In the present work, a laser-induced carbon (LIC) electrode is proposed for 4-Aminophenol (4-AP) electrochemical sensors. The electrode is patterned on a commercial low-cost polyimide (Kapton) sheet and functionalized with a multi-walled carbon nanotubes polyaniline (MWCNT-PANI) composite, realized by an in-situ-polymerization in an acidic medium. The LIC electrode modified with MWCNT-PAPNI nanocomposite was investigated by SEM, AFM, and electrochemically in the presence of ferri-ferrocyanide [Fe(CN)_6_]^3−/4−^ by cyclic voltammetry and impedance spectroscopy. The results show a significant improvement of the electron transfer rate after the electrode functionalization in the presence of the redox mediators [Fe(CN)_6_]^3−/4−^, related directly to the active surface, which itself increased by about 18.13% compared with the bare LIG. The novel electrode shows a good reproducibility and a stability for 20 cycles and more. It has a significantly enhanced electro-catalytic activity towards electrooxidation reaction of 4-AP inferring positive synergistic effects between carbon nanotubes and polyaniline PANI. The presented electrode combination LIC/MWCNT-PANI exhibits a detection limit of 0.006 μM for the determination of 4-AP at concentrations ranging from 0.1 μM to 55 μM and was successfully applied for the monitoring in real samples with good recoveries.

## 1. Introduction

Phenolic compounds (PCs) are acquiring exceptional attention due to their harmfulness to human body and to environment [1]. The European Chemicals Agency has placed them on the target List [2] under the REACH Legislation [2], and the US Environmental Protection Agency has set them on the List of Dangerous Contaminants [3].

4-Aminophenol (4-AP) has been detected as an intermediate substance for pharmaceutical preparation of the acetaminophen [4] and it can be obtained via the reduction of 4-nitrophenol pesticide [5]. 4-AP considered as a precursor for the synthesis of wide range of the materials for different application in various field, such as medical substances, rubber, dyestuff, feeding stuff and pesticides [6] and therefore, significant quantities of 4-AP can be predictably released as a pollutant into the environment. 4-AP is toxic and detrimental to human health by raising the body temperature for a long period [7]. Indeed, monitoring of 4-AP is essential and the development of low-cost, selective and sensitive analytical methods is urgently necessary.

The development of analytical electrochemistry has become a significant task of research in recent years [8]. In this context, several carbon-based electrodes have been proposed as transducer, primarily glassy carbon, carbon paste and screen-printed electrodes (SPE) [9]. Among them, graphene SPEs have been reported and well-characterized [10].

The graphite screen-printed electrodes are based on graphite ink and carbon black particles with a polymer as a binder, which is coated onto a substrate and then dried at a suitable temperature, which allows the mass production [11,12]. The commercially SPEs based on these materials need a standard micro-fabrication involving dip/spin-coating or screen-printing methods that are expensive or require additional binders or additives, which can be unfavourable for electrochemical [13,14]. However, the elaboration of complex solutions of carbon-based materials leads to clusters and aggregation due to the strong van-der-Waals interactions between sheets in graphene, significantly compromising the intrinsic high specific surface area and lessening the electrochemical activity [15]. The presence of organic solvents in the composite materials used for the functionalization leads to the destruction of the insulation inks, which reduces the sensitivity and the limit of detection [16,17]. Due to the low electron transfer rate, the functionalized electrodes become insensitive to several key analytes. The electroactive species in the samples can therefore easily disrupt SPCE-based biosensors [18].

In recent years laser induced graphene (LIG) has attracted a great attention for flexible electronics applications [19,20], especially in the field of electrochemical sensors [21,22,23]. In general, there are two type of interaction of laser and such materials, the photochemical and photothermal. In the case of photochemical, according to the literature, UV laser < 380 nm is required to obtain photochemical reaction or to use femtosecond lasers where the pulse duration is less the time of electron-hole recombination [24]. In the case of photothermal reaction to induce graphene structure on polyimide surface, the temperature has to reach certain amount to have a process called pyrolysis regardless of the laser wavelength [25]. Any type of laser, even the CO2 laser (10.6 µm) can achieve this process if the intensity and scanning speed are enough to escalate the temperature to burn the surface of the polymer. So different types of laser have been implemented in the literature to structure carboneous patterns on polyimide including the 405 nm continuous wave lasers. A CO_2_–laser-induced graphene on a Kapton surface shows an overall structure of porous graphene with a sheet structure with interconnected fibrous strands [26]. An induction by an UV laser displays micron-sized and nanometer pores with a decrease in nitrogen atoms. A Raman investigation with LIG on Kapton reports, that the 2D peak and the ratio 2D/G ratio show a better quality for CO_2_ LIG with a few layers of graphene [27].

In our previous works [28,29], 405 nm laser was used to directly pattern a commercial polyimide substrate into LIG. The results show that the structure of the induced carbon by this method is a graphene basal plane with a non-uniform scope of the oxygen-containing groups, coming from sp2 carbon clusters of some nanometers disconnected inside an imperfect carbon grid or a sp3 network.

However, the fabrication process of patterned carbon has several disadvantages, such as poor repeatability and the lack of control of the induced carbon film thickness. To overcome these limitations, the functionalization of laser-induced graphene electrodes surface has been proposed [30].

Several new materials have been used as electroactive for the electrochemical detection of 4-AP chemical compounds. In particular, Priya et al. reported the hydrothermal elaborated two-dimensional MoS_2_ modified glassy carbon electrode sensor to detect 4-AP in phosphate buffer solution. The fabricated sensor shows a detection limit of 2 nM in the linear range from 2 to 8 nM [31].

P. Shaikshavali et al. [32] succeeded in elaborating a hybrid nanocomposite based on CuO-Au decorated multiwalled carbon nanotubes (MWCNTs) and employed as sensitive material for the detection of 4-AP after its dispersion on the surface of glassy carbon electrode (GCE). The developed sensor showed a good linear response for 4-AP at the range of concentration from 0.5 μM to 1.6 μM with a detection limit of 0.105 μM. An electrochemical sensor for 4-AP monitoring based on graphene–chitosan composite film modified glassy carbon electrode (GCE) was fabricated by Huanshun Yin et al. [33]. Under the optimal experimental conditions, the oxidation peak current was proportional to 4-AP concentration in the range from 0.2 μM to 550 μM and 0.057 μM (S/N = 3) as a detection limit. Using the proposed sensor, 4-AP was successfully detected in water samples and paracetamol tablets. Qiuqun Liang et al. reported on the development of an electrochemical sensor based on cobalt complexes of Bis-Schiff bases to monitor 4-AP and paracetamol, which shows a linear LOD of 1.86 μM and 2.8 μM and a range of concentration from 5 to 30 μM [34]. Another method was developed by Nannan et al. combining silver, palladium nanoparticles to decorate reduced graphene oxide modified glassy carbon electrodes [35]. This strategy shows a low LOD of 0.013 μM in the linear range between 1 and 300 μM. All these investigations for 4-AP detection are reporting mainly on modified glassy carbon electrode (GCE), which are expensive [36] and need pretreatment before its functionalization [37]. For realizing such sensors electron-transfer and electroactivity become very important.

In the last decade conducting polymers have been used as a selective material for the electrochemical sensing application [38]. Among these polymers, polyaniline (PANI) has received widespread attention due to its low-cost, electrical conductivity (6.28 × 10^−9^ S·m^−1^), electrocatalytic activity and the easy fabrication process. PANI can be elaborated either electropolymerization or by chemical polymerization in acidic medium [39]. In addition, the development of PANI based nanocomposite has been a significant task of research specially its combination with carbon based materials or inorganic nanoparticles [40]. The MWCNT-PANI composite provides higher sensitivity compared to MWCNT and PANI as standalone.

In this work, 405 nm wavelength laser induced carbon electrode is used as a flexible electrode for the electrochemical detection of 4-AP. The LIC electrode was modified with in-situ polymerized MWCNT-PANI composite to reach higher sensitive, repeatable, and stable responses for the 4-Aminophenol chemical compound. For the detection of a 4-AP target, we prepare LIC/MWCNT-PANI electrodes and use the square wave voltammetry (SWV) method realizing a short response time.

## 2. Materials and Methods

Polyimide Kapton^®^ HN Film “thermal conductivity coefficient (0.12 W/m·K) and the refractive Index (sodium D line: 1.70)” was purchased from DuPont, 4-Aminophenol, standard phosphate buffer saline (PBS, pH = 7), ethanol, K_3_Fe(CN)_6_, K_4_Fe(CN)_6_, DMF, potassium peroxydisulfate (KPS), MWCNT, Aniline, hydrochloric acid (HCl), and potassium chloride (KCl) were purchased from Sigma-Aldrich and used without any further purification. The different pH of the PBS solution was adjusted using 1.0 M H_2_SO_4_. 4-AP solutions in water samples (contains 0.01 mg·mL^−1^) were prepared by adding the target without any prior treatment. In addition, for the paracetamol samples, two tablets (0.5 g/tablet contains 0.01 mg·mL^−1^ 4-AP) commercial ones were finely mortared to a powder. Then the powder was dissolved in 30 mL of anhydrous ethanol and stirred. Finally, the obtained solution was filtered after centrifugation for 5 min and diluted with anhydrous ethanol. The real samples measurements were carried out according to the standard addition method in tap water sample and paracetamol samples. UV-Vis spectrometer Perkin Elmer Norwalk CT 06859 USA Lamda 900 was used for the optical characterization of the prepared nanocomposites and FTIR spectroscopy type-Perkin Elmer model UATR two- Range between 400 and 4000 was used for the identification of chemical functional groups of the prepared nanocomposites. Impedance spectroscopy results were fitted by ZView software. For measurement of surface area and roughness, sensor were measure by (5600LS Atomic Force Microscope System AFM, Keysight Technologies, Santa Rosa, CA, USA) in tapping mode where using Pt conductive tip. All electrochemical measurements were carried out using PalmSens 4 potentiostat.

### 2.1. Fabrication of Laser-Induced Carbon Electrodes

**Figure 1:**
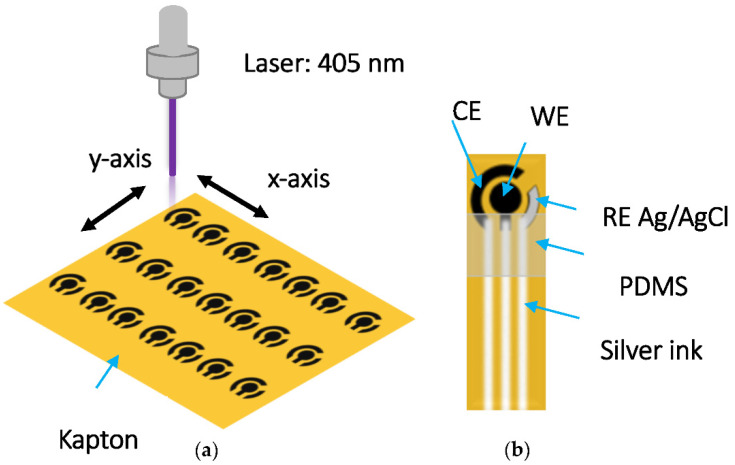
Schematic representation of (**a**) patterned electrodes and (**b**) prepared sensor.

LIC is formed by irradiating a polyimide (PI) Kapton HN substrate using set-up equipped with a visible laser source 405 nm wavelength operated in continuous mode at 2 W and an x-y motorized stage set at an exposure time of 40 ms (Figure 1a). The laser beam is adjusted in Y direction prior to irradiation such that the diameter of laser beam is 6 µm at optimal focal length 7 cm.

Upon laser writing, due to the photo-thermal effects, the orange-colored Kapton surface becomes transformed into a 3D pyrolytic carbon material is induced [25] (thickness > 10 μm) [41] (Figure S1). The contact was complemented with silver ink tracks to acquire a planer three-electrode sensor such as those of screen-printed electrodes. The working, counter and reference electrodes are highlighted in Figure 1b. The geometric area of the working electrode is 0.070 cm^2^. The reference electrode used here is Ag/AgCl. The main sensing area was isolated from the rest of the pattern by selective passivation using a polydimethylsiloxane (PDMS) hydrophobic coating.

### 2.2. Elaboration of PANI and MWCNT-PANI Composite

The polymerization of aniline is realized in an acidic solution, in a mutual process of elaboration. The process is as follow: a diluted KPS (20 mM 100 mL DI) solution was added drop-wise in 20 mL of aniline solution (2 mL aniline dissolved in 160 mL of 0.5 M chloric acid) under stirring for 12 h at 0–2 °C. At this stage, the precipitate was washed with chloric acid to remove the monomer until we reached a green color. Finally, the polymer was filtered and washed with DI to achieve pH 7 before being dried [42]. For the MWCN-PANI composite elaboration, 2 (wt %) weight percent of MWCNT relative to the nanocomposite MWCNT-PANI was sonicated in HCl (118 mL concentration 0.5 M) to obtain a homogeneous dispersion. After, 1.5 mL of aniline was added to the CNT solution and sonicated for 1 h. Then, under stirring, 4.32 g in 86 mL water of potassium persulfate was added drop-wise. The reaction was continued at 0–2 °C for 12 h. The dark green resulting product was filtered and washed.

### 2.3. Preparation of PANI/LIC Electrodes and MWCNT-PANI/LIC Electrodes

PANI and PANI-CNT nanocomposites were dissolved in DMF (1 mg/mL) and ultra-sonicated for 1 h. Three different electrodes were prepared: LIC, LIC/PANI and LIC/MWCNT-PANI. LIC/PANI was prepared by placing 2 μL of PANI solution into the surface of the LIC electrode. The obtained LIC/MWCNT-PANI was prepared by drop-casting PANI-CNT nanocomposite (1, 2 and 3 μL).

## 3. Results

### 3.1. Optical Performances of the Pristine Polyimide (PI) Sheet

**Figure 2:**
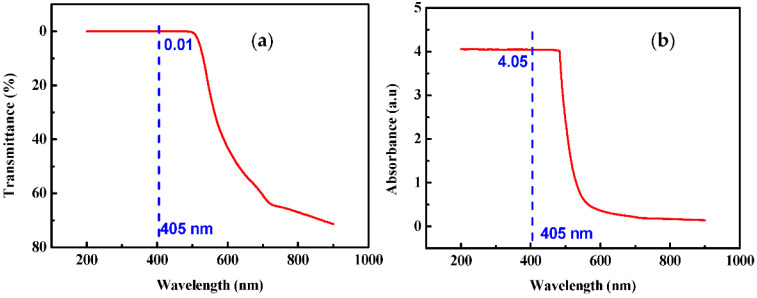
(**a**) Red solid line: transmission spectrum and (**b**) red solid line: absorption spectrum of pristine polyimide (blue dotted line in each curve shows the guidance to the transmission and absorption at 405 nm).

Figure 2 shows the UV-vis transmission and absorption spectrums of pristine PI film, indicating that the PI film has a poor transmission (0.01%) at 405 nm and a strong absorption (4.05 a.u) for UV laser.

### 3.2. Characterization of PANI and MWCNT-PANI Composite

**Figure 3:**
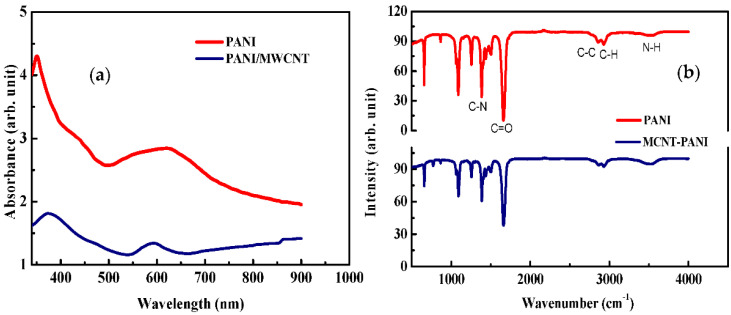
(**a**) UV−Visible of PANI and PANI/MWCNT in DMF and (**b**) Infrared spectroscopy of PANI and MWCNT−PANI in DMF.

The UV-Vis results of PANI and MWCNT-PANI in DMF solution are presented in Figure 3a. Two peaks are obtained with PANI. The first peak in the UV wavelength range between 340 and 360 nm is related to π−π* excitation and the second peak in the visible wavelength range is related to the quinone rings exciton present in PANI. The dark green coloration of the obtained PANI and the two peaks obtained in the UV spectrum indicate the emeraldine salt form of polyaniline [43,44]. The UV-vis spectra of MWCNT-PANI nanocomposites (blue) also show the presence two absorption bands. Due to the overlap between PANI and MWCNTs, the π−π* band has been red shifted. The UV region band is shifted to the higher wavelength side due to the addition of MWCNTs. This indicates the good distribution of the MWCNTs among the PANI [45]. This phenomenon increases the interaction energy between PANI and MWCNTs (as stated in the FTIR spectrum). The improved absorption of MWCNT-PANI composite compared to PANI can be attributed to the formation of a higher number of polarons and bipolarons as reported in the previous works [46], which has been confirmed from the amelioration of the electrical conductivity along with the interaction between PANI quinoid rings and MWCNT. Based on the Tauc relation in Equation (1), the prepared PANI and MWCNT-PANI composites’ optical band gap was calculated. The power coefficient n determined the type of possible electronic transitions during absorption processes [47]. n = ½ for the direct band transition of the nanocomposites. The direct band gap was obtained from extrapolating the straight portion of the plot on hν axis at α = 0. The obtained band gaps E_g_ 2.54 eV for PANI and 2.40 eV for MWCNT-PANI. These values are in good agreement with the results found in [46]. Where, E_g_ is the optical band gap, α is the absorption coefficient, ϑ is the frequency and A is a constant.

αhϑ = A(hϑ − E_g_)^n^, (1)

Infrared spectroscopy is a valuable method to characterize the interactions between MWCNT and PANI Figure 3b. It exhibits the clear presence of benzoid C−N at 1262 cm^−1^ and the C=N quinoid ring vibration at 1506 cm^−1^ [48] indicating the oxidation state of emeraldine salt of PANI. Very weak and broad band around 3000 cm^–1^ is assigned to the NH stretching mode at 3500 cm^−1^ [49]. The band at 2930 cm^−1^ corresponds to C-H bending [44]. By adding 0.2 wt % of MWCNT into the PANI matrix, the characteristic features of MWCNT are not observed. In contrast, the main features of the PANI were maintained, which indicates that the MWCNT was well interpenetrated into the PANI matrix, as well as the decrease in the absorption. A small peak was found at 776 cm^−1^, which can be explained as a new form of C-H out of plan bending maybe due to the chemical interactions of PANI and MWCNT.

### 3.3. Surface Area A

**Figure 4:**
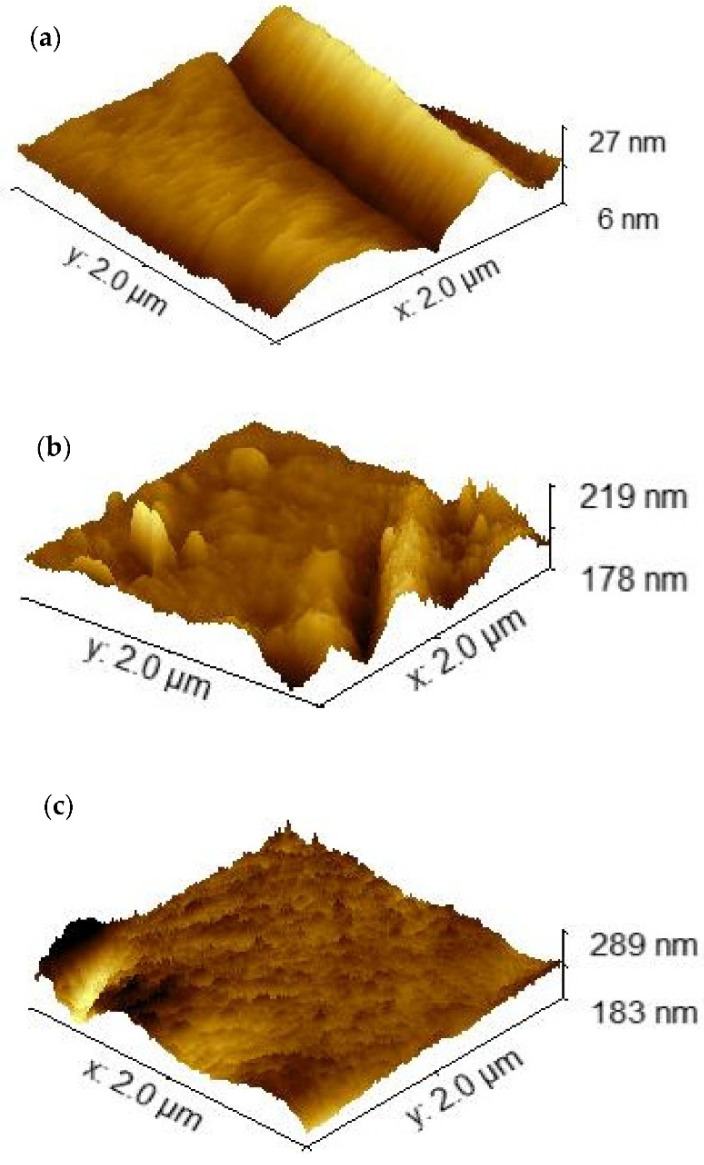
AFM images of (**a**) LIC, (**b**) LIC/PANI and (**c**) LIC/MWCNT-PANI.

There are several concerns about using the Randles-Sevcik equation to estimate the electrode surface area, mainly when nanomaterials are being used for electrode modification [50]. For determining the surface area of the different electrodes LIC, LIC/PANI and LIC/MWCNT-PANI we use atomic force microscopy (AFM) [51,52]. The roughness of the three electrodes samples has been determined for a 4 μm^2^ representative area. Figure 4 shows the 3D projections of the AFM. The surface area A and the roughness are determined using WSxM software [53] and listed in Table 1. The larger surface area is achieved with LIC/MWCNT-PANI, for which the surface A increased by about 18.13% compared with the flat one. The SEM (Figure S2) images clearly show a well-dispersed MWCNT-PANI composite with slight agglomerations. The polymeric materials well coated the CNTs bundles, which matches well with previous published reports [48,54]. This type of morphology provides a large surface area for the interaction with 4-AP.

### 3.4. Electrochemical Investigation of 4-AP

**Figure 5:**
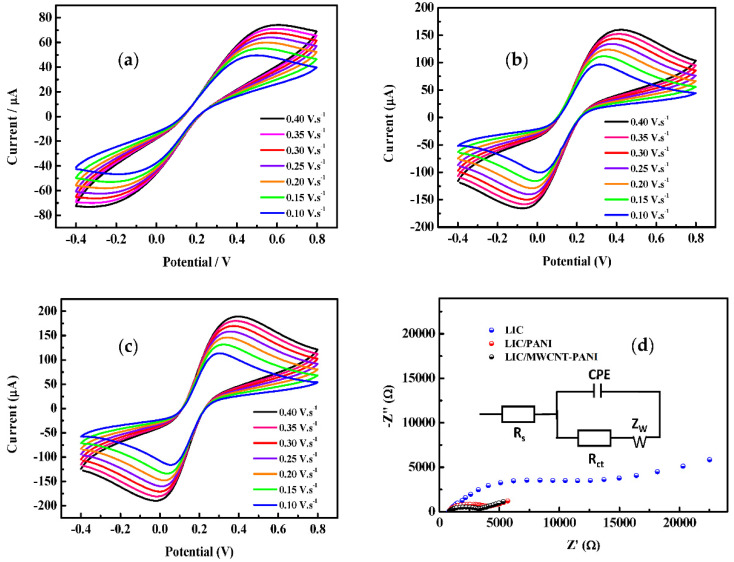
CVs of the prepared transducers, (**a**) LIC, (**b**) LIC/PANI and (**c**) LIC/MWCNT-PANI recorded at different scan rates from 100 to 400 mV·s^−1^ in the presence of the redox mediators 5 mM [Fe(CN)_6_]^3−/4−^ in 0.1 M KCl and (**d**) Nyquist plots of the bare LIC, LIC/PANI and LIC/MWCNT-PANI electrodes; inset: equivalent circuit.

The transfer process at the surface of the prepared electrodes was studied. CVs of the different prepared electrodes were recorded at different scan rates ranging from 100 to 400 mV·s^−1^ in the presence of the redox mediators 5.0 mM [Fe(CN)_6_]^3−/4−^ in 0.1 M KCl as a supporting electrolyte as presented in (Figure 5). The results present a quasi-reversible reaction behavior of [Fe(CN)_6_]^3−/4−^ for all the electrodes. In addition, a linear increase of the current against the square root of the scan rate, indicates that the voltammetric response was controlled by diffusion process [55]. Interestingly, the oxidation and reduction current peaks obtained for LIC/MWCNT-PANI increased about 61% and 18% compared to the bare LIG and LIC/MWCNT-PANI (2 μL) electrodes, respectively. The role played by MWCNT-PANI composite in the modification of the LIG electrode was studied further by EIS. The Nyquist plots were presented in Figure 5d. The EIS includes a semicircular part and a linear part [56]. At higher frequencies, the semicircular corresponds to the electron transfer limited process and the diameter is equivalent to the charge transfer resistance (R_ct_). While the linear part at lower frequencies represents the diffusion process. The related electrical equivalent circuit diagram is shown in Figure 5d inset. The EIS data were fitted by Zview software. According to the fitted EIS results, the impedance decreases significantly from bare to modified LIC, with LIC/MWCNT-PANI displaying the lowest R_ct_. It is observed that for bare LIC, the value of R_ct_ is the highest (R_ct_ = 9.200 ± 0.036 KΩ), whereas for LIC modified by PANI, the R_ct_ values are intermediary (R_ct_ = 2.464 ± 0.021 KΩ). The smallest value of R_ct_ have been reached for LIC/MWCNT-PANI (R_ct_ = 2.200 ± 0.016 KΩ). Therefore, MWCNT-PANI enlarges the surface area, as measured by AFM and decreases the charge-transfer resistance at the electrode-electrolyte interface. The Nyquist diagram analysis is consistent with CV measurement and further proved the successful construction of MWCNT-PANI based composite for electrochemical sensor [56].

For further investigation, the electron transfer ET rate was estimated based on cyclic voltammetry results. The CVs in Figure 5 revel quasi-reversible nature of a reactions which results in an increase of the peak-to-peak separation ($\Delta E_{P}$ by increasing the scan rate (*v*). In this case the ($k_{0}$ heterogeneous ET rate can be calculated, using the method developed by Klingler and Kochi Equation (2) [57].

(2) $$k_{0}=2.18{\left(\frac{\alpha nFDv}{RT}\right)}^{1/2}e^{-[(\alpha^{2}F/RT)n∆E_{P}]\;},$$

*α* is the transfer coefficient = 0.5,
*n* is the number of electrons transferred,
*F* is the Faraday constant, and
*R* and *T* have their usual meanings.
*D* diffusion coefficients of the oxidized and reduced form.
*ν* scan rate.
$\Delta E_{P}$ peak-to-peak separation.

The $k_{0}$ was calculated for each scan rate and the arithmetic means obtained as shown in Figure S3. It was found that the ET rate $k_{0}$ frequently changed altogether over the surface of the LIC after the modification with PANI and MWCNT-PANI nanocomposite samples. The LIC/MWCNT-PANI electrode presents the faster transfer kinetics which can be attributed to the large specific area and good electroactivity of MWCNT-PANI nanocomposite.

**Figure 6:**
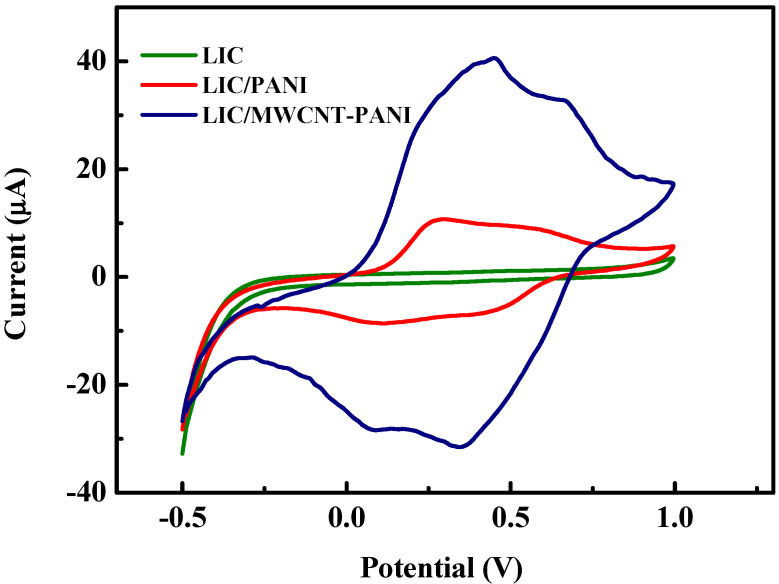
CV curves of prepared electrodes in 1 M HCl scan rate 50 mV·s^−1^.

In order to investigate the electro-catalytic activity of the PANI and MWCNT-PANI films at the electrode surface, CV measurements were carried in 0.1 M HCl (pH = 1) from −0.5 to 1 V at a scan rate of 50 mV·s^−1^ (Figure 6). The typical CV response of polyaniline has been well reported in previous works [58] showing two sets of oxido-reduction peaks. The first set of redox peaks is linked to the conversion of the reduced leucoemeraldine base to the partially oxidized emeraldine. The second is related to the conversion of emeraldine to the fully oxidized pernigraniline. At the bare LIC, no significant electrocatalytic current response has been observed. The difference in electrochemical behavior between the pure PANI and MWCNT-PANI nanocomposite films is clear from the CVs.

The MWCNT-PANI film demonstrates the higher current peaks, explain the improved electroactivity compared pure PANI.

### 3.5. Electrochemical Detection of 4-AP

**Figure 7:**
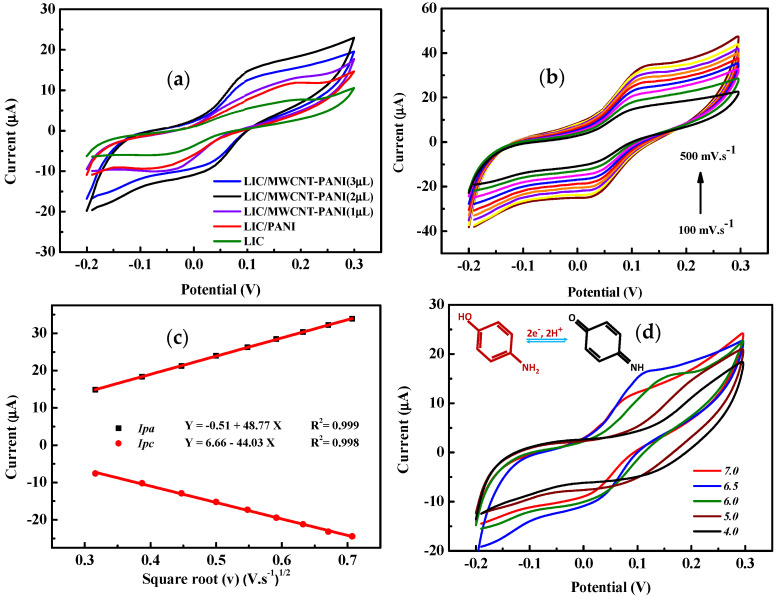
(**a**) CVs obtained by bare LIC, LIC/PANI and LIC/MWCNT-PANI in the presence of 1.0 mM 4-AP in 0.1 M PBS (pH = 6.5) Scan rate of 100 mV·s^−1^, (**b**) LIC/MWCNT-PANI in the presence of 1.0 mM 4-AP in 0.1 M PBS (pH = 6.5) at different scan rates from 100 mV·s^−1^ to 500 mV·s^−1^; (**c**) Linear fitting of the peak current with the square root of scan rate. Inset oxidation current against the scan rate (100 to 500 mV·s^−1^); (**d**) CVs LIC/MWCNT-PANI response of 1 mM 4-AP in 0.1 M PBS in different at 100 mV·s^−1^ scan rate and the inset: Redox reaction mechanism of 4-AP.

The electrochemical behavior of 1 mM of 4-AP was investigated with the different prepared electrodes (LIC and LIC/PANI and LIC/MWCNT-PANI (1, 2 and 3 μL)). The CVs were recorded in 1 mM 4-AP 0.1 M phosphate buffer solution pH = 6.5 shown in Figure 7a. The results show a quasi-reversible redox peak in all cases. Where the effect of the combination of carbon nanotubes and polyaniline is seen. It is noted that the highest current among the different electrodes is obtained after the modification with MWCNT-PANI (2 μL) and it is about 2 and 1.4 times higher than that of LIC and LIC/PANI. The higher LIC/MWCNT-PANI electrode sensitivity towards 4-AP can be attributed to the adsorption ability (porous surfaces in LIC/MWCNT-PANI electrode), the high catalytic activity of the conductive nanocomposite due to the synergistic effect between polyaniline and carbon nanotubes. The electrochemical behavior of 4-AP at the surface of nanocomposite modified LIC electrode is well documented in previous reports [4,31]. At the surface of MWCNT-PANI film, the electrons are released in the presence of 4-AP, which improve and enhance the electrochemical reaction during the measurement.

Useful information concerning the electrochemical reaction mechanisms can be obtained from the potential scan rate. Therefore, the electrochemical response of 4-AP (1 mM) in 0.1 M phosphate buffer (pH = 6.5) was investigated at different scan rates from 100 mV·s^−1^ to 500 mV·s^−1^ by cyclic voltammetry Figure 7b. The results illustrated a linear regression current against the square root of the scan rate Figure 7c, which is a characteristic diffusion-controlled process for the 4-AP oxidation [59].

The pH effect towards the electrochemical response of 4-AP was inspected in PBS using CV technique at the surface of LIC/MWCNT-PANI electrode. The CV responses of 1 mM 4-AP were carried out in the range of 4.0 to 7.0 in order to pick the best pH for the sensor operating point as presented in Figure 7d. From the voltammograms behavior, the Ip_a,c_ of 4-AP increased from pH 4.0 to reach the well-defined highest peak current at pH = 6.5 and then decreased. Therefore, pH 6.5 was selected as a supporting electrolyte in all the experiments for the determination of 4-AP. On increasing the pH of PBS solution, the oxidation peak potentials of 4-AP shifted towards the negative potential, which indicates the involvement of the protons in the electrochemical oxidation of 4-AP at LIC/MWCNT-PANI electrode [32]. The possible redox reaction mechanism is presented in inset Figure 7d.

### 3.6. Dtermination of 4-AP by SWV

#### 3.6.1. Analytical Curve

The high sensitivity of LIC/MWCNT-PANI provides an efficient electron interaction, which improves the direct electron transfer between MWCNT-PANI and LICE active sites. The LIC/MWCNT-PANI sensor provided a simple and reliable method for hazardous chemical detection. It has been also demonstrated as a sensitive material for the monitoring of several chemicals in the environmental and health-care sectors. Table 2 shows some selected sensors based on carbon nanotubes/polyaniline nanocomposite for different chemical compounds with various analytical methods. Most of them are using fluorescence, amperometry or differential pulse voltammetry as detection method.

**Figure 8:**
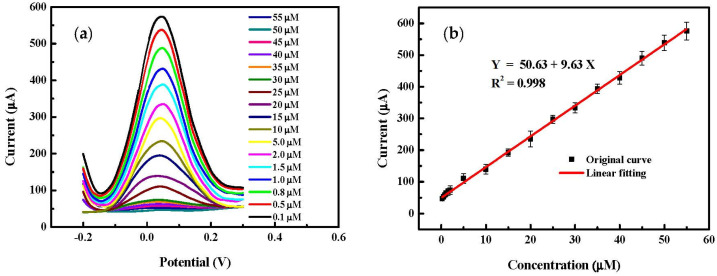
(**a**) SWV of LIC/MWCNT-PANI electrode with variation of the concentration from 0.1 μM to 55 μM and (**b**) Calibration curve: Linear fitting of the peak current with the 4-AP concentration.

The SWV technique is increasingly used in the field of chemical sensors because of its high sensitivity due to the ability to discriminate the capacitive current [60,61,62]. It has been used here to detect 4-AP with the proposed electrode (LIC/MWCNT-PANI). The effects of frequency from 1.0 to 20 Hz at fixed pulse amplitude (0.1 V) and potential step (0.01 V), on the LIC/MWCNT-PANI sensor response to 1.0 μM 4-AP in PBS (pH = 6.5) solution were studied (Figure S4). The highest and the stable analytical signal was obtained at 5 Hz, thus this frequency was selected for the sensor calibration. While the detection was performed in the possible range from −0.2 to 0.3 V and the pH test medium was adjusted to 6.5, the SWV responses were recorded with different concentrations of 4-AP from 0.1 μM to 55 μM (Figure 8a). It can be seen from the calibration curve in (Figure 8b) that a quite linear variation of the current can be observed with the increase of the concentration in the selected range Ip_a_ (μA) = 50.63 + 9.63 × [4-AP], with a detection limit of 0.006 μM. The limit of detection has been determined based on Equation (3).

(3) $$LOD=\frac{3S}{m}\;,$$

where, (*S*) is the relative standard deviation of the blank analyte signal and (*m*) is the slope of the calibration curve.

The limit of detection of the proposed sensor is lower than several previously reported results based on nano-composite and nano-materials modified electrodes (Table 2). The value found is better than that obtained with other electrodes reported in the literature and briefly summarized in Table 2. MWCNT-PANI provides a high sensitivity and a good electroactivity for 4-AP detection due to its high specific surface area.

**Table 2:** Performance parameters of different electrochemical sensor methods for 4-AP detection.

Electrode	LOD (μM)	Range (μM)	References
Au/Pd/rGO/GCE	0.12	1–300	[6]
Cu-Au MWCNT nanocomposite/GCE	0.105	0.5–1.6	[32]
Graphene chitosan/GCE	0.057	0.2–550	[33]
Bis-schiff Base Cobalt Complexes/GCE	2.08	5–30	[34]
AuNPs and a Layered Double Hydroxide Sodium/GCE	0.1	0.5–400	[63]
Graphene-Polyaniline	15.68	50–500	[64]
Fc-PAA-GNPs/GCE	7.61	30–1064	[65]
LIC/MAWCNT-PANI	0.006	0.1–55	This work

#### 3.6.2. Reproducibility, Stability and Selectivity

**Figure 9:**
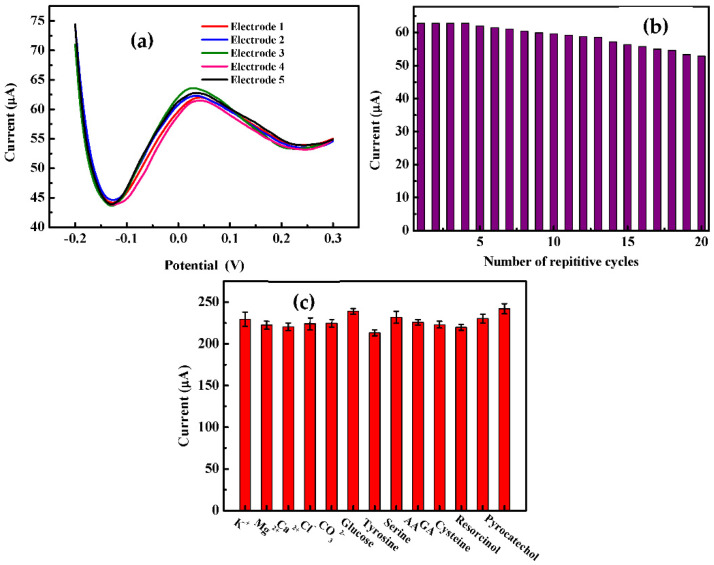
(**a**) CVs of five electrodes prepared independently for measuring 1.0 μM 4-AP; (**b**) 20 cycles of SWV were carried using 1 μM 4-AP solution in PBS pH 6.5 at LIC/MWCNT-PANI and (**c**) Anodic current for 4-AP oxidation measured in the presence of 100-fold excess of interfering ions at a 4-AP concentration of 10 μM (pH = 6.5), and SWV under following conditions: 5 Hz, potential step 0.01, amplitude 0.1 V.

The relative standard deviations (RSD) of five electrodes prepared separately for measuring 1.0 μM 4-AP is determined to be 3.17% to test the modified electrode’s fabrication reproducibility (Figure 9a). The results suggest a good fabrication reproducibility. Further, to demonstrate the stability of the LIC/MWCNT-PANI electrode 20 cycles of SWV were carried using 1 μM 4-AP solution in PBS pH 6.5 at the proposed electrode (Figure 9b). The experimental results show the fabrication protocol is reproducible.

The influence of some inorganic ions, organic compounds and other phenolic compounds on the determination of 4-AP was studied and the results are presented in Figure 9c. The results show that 100-fold of K^+^, Mg^2+^, Ca^2+^, Cl^−^, CO_3_^2−^, glucose, tyrosine, serine and ascorbic acid (AA), gallic acid (GA), cysteine, resorcinol and pyrocatechol do not interfere with the oxidation signal of 20 μM 4-AP.

#### 3.6.3. Determination of 4-AP in Real Samples

In order to assess the practical application of the proposed method, the LIC/MWCNT-PANI electrode was used to determine 4-AP in tap water samples and paracetamol tablet samples using the standard addition method (Figure S5). All the measurements were repeated three times under the same conditions. Table 3 and Table 4 present the results. The recovery of 4-AP, compared to the same concentration in PBS, was in the range from 96.4% to 105.41%, revealing that this method is effective and reliable.

## 4. Conclusions

A flexible, sensitive sensor for 4-AP electrochemical detection was developed in this work using MWCNT-PANI nanocomposite elaborated by the in-situ polymerization method in acidic medium, modified laser-induced carbon LIC/MWCNT-PANI. The prepared nanocomposite was characterized by UV-vis, FTIR and CV in HCl. The preparation of the electrodes is performed by a simple and easy method of deposition. An improvement of the current peaks by cyclic voltammetry and increase of the conductivity by evaluation of charge transfer resistance suggest the synergetic effect between MWCNTs and PANI with the excellent conductivity and large specific surface area. The LIC/MWCNT-PANI sensor was tested for the detection of 4-Aminophenol and it was found to have a detection limit of 0.006 μM. The work demonstrates the feasibility of fabricating robust and cost-effective electrochemical sensors by laser patterning on polyimide films, suitable for the detection of 4-AP.

## Figures and Tables

**Table 1:** Surface area and roughness of LIC, LIC/PANI and LIC/MWCNT-PANI.

Electrodes	Surface Area (μm^2^)	Roughness (nm)
LIC	4.08 ± 0.49	2.58 ± 0.31
LIC/PANI	4.16 ± 0.48	2.88 ± 0.33
LIC/MWCNT-PANI	4.82 ± 0.48	4.96 ± 0.51

**Table 3:** Determination of 4-AP in water samples.

Samples	Added Concentration (μM)	Current (µA)	RSD (%) (*n* = 3)	Recovery (%)
1	5	108.22	3.03	97.63
2	10	134.75	2.71	96.40
3	15	188.75	2.86	98.17

**Table 4:** Determination of 4-AP in paracetamol tablet samples.

Samples	Added Concentration (μM)	Current (µA)	RSD (%) (*n* = 3)	Recovery (%)
1	5	113.06	2.81	102.00
2	10	135.60	3.42	100.63
3	15	198.97	3.11	105.41

## Data Availability

Not applicable.

## Supplementary Materials

The following are available online at https://www.mdpi.com/article/10.3390/s22030833/s1, Figure S1: Raman spectrum of laser induced carbon, Figure S2: SEM of LIC/MWCNT-PANI, Figure S3: Klingler-Kochi analyses of the calculated ET rates ($k_{0}$) for each scan rate, Figure S4: SWV of 1.0 μM 4-AP in PBS (pH = 6.5) at different frequencies from 1.0 Hz to 20 Hz Figure S5: (a) SWV in water samples and (b) in Paracetamol samples.

## References

[1] Della Pelle F., Compagnone D. (2018). Nanomaterial-Based Sensing and Biosensing of Phenolic Compounds and Related Antioxidant Capacity in Food. Sensors.

[2] Substance Information—ECHA. https://echa.europa.eu/substance-information/-/substanceinfo/100.003.303.

[3] US EPA Water Quality Standards Regulations: New York. https://www.epa.gov/wqs-tech/water-quality-standards-regulations-new-york.

[4] Vilian A.T.E., Veeramani V., Chen S.-M., Madhu R., Huh Y.S., Han Y.-K. (2015). Preparation of a Reduced Graphene Oxide/Poly-l -Glutathione Nanocomposite for Electrochemical Detection of 4-Aminophenol in Orange Juice Samples. Anal. Methods.

[5] Tang Y., Huang R., Liu C., Yang S., Lu Z., Luo S. (2013). Electrochemical Detection of 4-Nitrophenol Based on a Glassy Carbon Electrode Modified with a Reduced Graphene Oxide/Au Nanoparticle Composite. Anal. Methods.

[6] Wang H., Zhang S., Li S., Qu J. (2018). Electrochemical Sensor Based on Palladium-Reduced Graphene Oxide Modified with Gold Nanoparticles for Simultaneous Determination of Acetaminophen and 4-Aminophenol. Talanta.

[7] Li G., Sun P., Wu F., Zhao J., Han D., Cui G. (2020). Significant Enhancement in the Electrochemical Determination of 4-Aminophenol from Nanoporous Gold by Decorating with a Pd@CeO_2_ Composite Film. New J. Chem..

[8] El Harrad L., Bourais I., Mohammadi H., Amine A. (2018). Recent Advances in Electrochemical Biosensors Based on Enzyme Inhibition for Clinical and Pharmaceutical Applications. Sensors.

[9] Griffiths K., Dale C., Hedley J., Kowal M.D., Kaner R.B., Keegan N. (2014). Laser-Scribed Graphene Presents an Opportunity to Print a New Generation of Disposable Electrochemical Sensors. Nanoscale.

[10] Randviir E.P., Brownson D.A.C., Metters J.P., Kadara R.O., Banks C.E. (2014). The Fabrication, Characterisation and Electrochemical Investigation of Screen-Printed Graphene Electrodes. Phys. Chem. Chem. Phys..

[11] Metters J.P., Kadara R.O., Banks C.E. (2011). New Directions in Screen Printed Electroanalytical Sensors: An Overview of Recent Developments. Analyst.

[12] Honeychurch K.C. (2012). Screen-Printed Electrochemical Biosensors and Sensors for Monitoring Metal Pollutants. Insci. J..

[13] Li W., Tan C., Lowe M.A., Abruña H.D., Ralph D.C. (2011). Electrochemistry of Individual Monolayer Graphene Sheets. ACS Nano.

[14] Jakus A.E., Secor E.B., Rutz A.L., Jordan S.W., Hersam M.C., Shah R.N. (2015). Three-Dimensional Printing of High-Content Graphene Scaffolds for Electronic and Biomedical Applications. ACS Nano.

[15] Zhang Q., Zhang F., Medarametla S.P., Li H., Zhou C., Lin D. (2016). 3D Printing of Graphene Aerogels. Small.

[16] Del Carlo M., Di Marcello M., Perugini M., Ponzielli V., Sergi M., Mascini M., Compagnone D. (2008). Electrochemical DNA Biosensor for Polycyclic Aromatic Hydrocarbon Detection. Microchim. Acta.

[17] Lucarelli F., Authier L., Bagni G., Marrazza G., Baussant T., Aas E., Mascini M. (2003). DNA Biosensor Investigations in Fish Bile for Use as a Biomonitoring Tool. Anal. Lett..

[18] Malhotra B.D., Chaubey A. (2003). Biosensors for Clinical Diagnostics Industry. Sens. Actuators B Chem..

[19] Ye R., James D.K., Tour J.M. (2019). Laser-Induced Graphene: From Discovery to Translation. Adv. Mater..

[20] Ye R., James D.K., Tour J.M. (2018). Laser-Induced Graphene. Acc. Chem. Res..

[21] de Araujo W.R., Frasson C.M.R., Ameku W.A., Silva J.R., Angnes L., Paixão T.R.L.C. (2017). Single-Step Reagentless Laser Scribing Fabrication of Electrochemical Paper-Based Analytical Devices. Angew. Chem. Int. Ed..

[22] Mendes L.F., de Siervo A., Reis de Araujo W., Longo Cesar Paixão T.R. (2020). Reagentless Fabrication of a Porous Graphene-like Electrochemical Device from Phenolic Paper Using Laser-Scribing. Carbon.

[23] Kim J.D., Kim T., Pak J. (2018). Fabrication and Transfer of Laser Induced Graphene (LIG) Electrode for Flexible Substrate-Based Electrochemical Sensor Applicatins. Trans. Korean Inst. Electr. Eng..

[24] Wang L., Wang Z., Bakhtiyari A.N., Zheng H. (2020). A Comparative Study of Laser-Induced Graphene by CO2 Infrared Laser and 355 Nm Ultraviolet (UV) Laser. Micromachines.

[25] Mamleyev E.R., Heissler S., Nefedov A., Weidler P.G., Nordin N., Kudryashov V.V., Länge K., MacKinnon N., Sharma S. (2019). Laser-Induced Hierarchical Carbon Patterns on Polyimide Substrates for Flexible Urea Sensors. NPJ Flex. Electron..

[26] Getachew B.A., Bergsman D.S., Grossman J.C. (2020). Laser-Induced Graphene from Polyimide and Polyethersulfone Precursors as a Sensing Electrode in Anodic Stripping Voltammetry. ACS Appl. Mater. Interfaces.

[27] Ma W., Zhu J., Wang Z., Song W., Cao G. (2020). Recent Advances in Preparation and Application of Laser-Induced Graphene in Energy Storage Devices. Mater. Today Energy.

[28] Nasraoui S., Al-Hamry A., Teixeira P.R., Ameur S., Paterno L.G., Ben Ali M., Kanoun O. (2021). Electrochemical Sensor for Nitrite Detection in Water Samples Using Flexible Laser-Induced Graphene Electrodes Functionalized by CNT Decorated by Au Nanoparticles. J. Electroanal. Chem..

[29] Nasraoui S., Al-Hamry A., Anurag A., Teixeira P.R., Ameur S., Paterno L.G., Ben Ali M., Kanoun O. Investigation of Laser Induced Graphene Electrodes Modified by MWNT/AuNPs for Detection of Nitrite. Proceedings of the 2019 16th International Multi-Conference on Systems, Signals & Devices (SSD).

[30] Lin X., Lu Z., Dai W., Liu B., Zhang Y., Li J., Ye J. (2018). Laser Engraved Nitrogen-Doped Graphene Sensor for the Simultaneous Determination of Cd(II) and Pb(II). J. Electroanal. Chem..

[31] Ramasubramanian P.A., Thangavel S., Nallamuthu G., Kirabakaran K., Vasudevan V., Ravichandran K., Venugopal G. (2018). A Novel MoS2 Structures for Electrochemical Detection of 4-Aminophenol. J. Mater. Sci. Mater. Electron..

[32] Shaikshavali P., Madhusudana Reddy T., Palakollu V.N., Karpoormath R., Subba Rao Y., Venkataprasad G., Gopal T.V., Gopal P. (2019). Multi Walled Carbon Nanotubes Supported CuO-Au Hybrid Nanocomposite for the Effective Application towards the Electrochemical Determination of Acetaminophen and 4-Aminophenol. Synth. Met..

[33] Yin H., Ma Q., Zhou Y., Ai S., Zhu L. (2010). Electrochemical Behavior and Voltammetric Determination of 4-Aminophenol Based on Graphene–Chitosan Composite Film Modified Glassy Carbon Electrode. Electrochim. Acta.

[34] Liang Q., Liu Z., Liang C., Han G., Zhang S., Feng X. (2019). Electrochemical Simultaneous Detection of Paracetamol and 4- Aminophenol Based on Bis-Schiff Base Cobalt Complex. Int. J. Electrochem. Sci..

[35] Dou N., Zhang S., Qu J. (2019). Simultaneous Detection of Acetaminophen and 4-Aminophenol with an Electrochemical Sensor Based on Silver–Palladium Bimetal Nanoparticles and Reduced Graphene Oxide. RSC Adv..

[36] Sharma S. (2018). Glassy Carbon: A Promising Material for Micro- and Nanomanufacturing. Materials.

[37] Anodic Pretreatment of Glassy Carbon: Impacts on Structural and Electrochemical Characteristics of Niox Nanoparticles—MedCrave Online. https://medcraveonline.com/IJBSBE/anodic-pretreatment-of-glassy-carbon-impacts-on-structural-and-electrochemical-characteristics-of-niox-nanoparticles.html.

[38] Naveen M.H., Gurudatt N.G., Shim Y.-B. (2017). Applications of Conducting Polymer Composites to Electrochemical Sensors: A Review. Appl. Mater. Today.

[39] Gospodinova N., Terlemezyan L. (1998). Conducting Polymers Prepared by Oxidative Polymerization: Polyaniline. Prog. Polym. Sci..

[40] Essousi H., Barhoumi H., Bibani M., Ktari N., Wendler F., Al-Hamry A., Kanoun O. (2019). Ion-Imprinted Electrochemical Sensor Based on Copper Nanoparticles-Polyaniline Matrix for Nitrate Detection. J. Sens..

[41] Stanford M.G., Zhang C., Fowlkes J.D., Hoffman A., Ivanov I.N., Rack P.D., Tour J.M. (2020). High-Resolution Laser-Induced Graphene. Flexible Electronics beyond the Visible Limit. ACS Appl. Mater. Interfaces.

[42] Rahman M.M., Hussein M.A., Alamry K.A., Al-Shehry F.M., Asiri A.M. (2018). Polyaniline/Graphene/Carbon Nanotubes Nanocomposites for Sensing Environmentally Hazardous 4-Aminophenol. Nano-Struct. Nano-Objects.

[43] Mazzeu M.A.C., Faria L.K., Cardoso A.D.M., Gama A.M., Baldan M.R., Gonçalves E.S. (2017). Structural and Morphological Characteristics of Polyaniline Synthesized in Pilot Scale. J. Aerosp. Technol. Manag..

[44] Karbownik I., Rac-Rumijowska O., Fiedot-Toboła M., Rybicki T., Teterycz H. (2019). The Preparation and Characterization of Polyacrylonitrile-Polyaniline (PAN/PANI) Fibers. Materials.

[45] Abdulla S., Mathew T.L., Pullithadathil B. (2015). Highly Sensitive, Room Temperature Gas Sensor Based on Polyaniline-Multiwalled Carbon Nanotubes (PANI/MWCNTs) Nanocomposite for Trace-Level Ammonia Detection. Sens. Actuators B Chem..

[46] Rasheed H.K., Kareem A.A. (2018). Effect of Multiwalled Carbon Nanotube Reinforcement on the Opto-Electronic Properties of Polyaniline/c-Si Heterojunction. J. Opt. Commun..

[47] Das A.K., Bhowmik R., Meikap A.K. (2017). Surface Functionalized Carbon Nanotube with Polyvinylidene Fluoride: Preparation, Characterization, Current-Voltage and Ferroelectric Hysteresis Behaviour of Polymer Nanocomposite Films. AIP Adv..

[48] Sreekala P.S., John H., Aanandan C.K. (2020). Studies on Anomalous Dispersion Behavior of PANI–CNT Composites for Enhanced Shielding Effectiveness in Various Microwave Bands. Appl. Phys. A.

[49] Halvaee M., Didehban K., Goodarzi V., Ghaffari M., Ehsani M., Saeb M.R. (2017). Comparison of Pristine and Polyaniline-Grafted MWCNTs as Conductive Sensor Elements for Phase Change Materials: Thermal Conductivity Trend Analysis. J. Appl. Polym. Sci..

[50] Paixão T.R.L.C. (2020). Measuring Electrochemical Surface Area of Nanomaterials versus the Randles−Ševčík Equation. ChemElectroChem.

[51] Wei C., Sun S., Mandler D., Wang X., Qiao S.Z., Xu Z.J. (2019). Approaches for Measuring the Surface Areas of Metal Oxide Electrocatalysts for Determining Their Intrinsic Electrocatalytic Activity. Chem. Soc. Rev..

[52] Sharifi-viand A., Mahjani M.G., Jafarian M. (2014). Determination of Fractal Rough Surface of Polypyrrole Film: AFM and Electrochemical Analysis. Synth. Met..

[53] Horcas I., Fernández R., Gómez-Rodríguez J.M., Colchero J., Gómez-Herrero J., Baro A.M. (2007). WSXM: A Software for Scanning Probe Microscopy and a Tool for Nanotechnology. Rev. Sci. Instrum..

[54] Abdel-Gaber A.M., Abd-El-Nabey B.A., Khamis E., Salman R.M., Rahal H.T., El Morr Z. (2020). Electrochemical Synthesis and Corrosion Behaviour of Polyaniline on Stainless Steel in Sodium Hydroxide Solutions. Chem. Eng. Commun..

[55] García-Miranda Ferrari A., Foster C., Kelly P., Brownson D., Banks C. (2018). Determination of the Electrochemical Area of Screen-Printed Electrochemical Sensing Platforms. Biosensors.

[56] Niu X., Yang X., Li H., Shi Q., Wang K. (2021). Chiral Voltammetric Sensor for Tryptophan Enantiomers by Using a Self-Assembled Multiwalled Carbon Nanotubes/Polyaniline/Sodium Alginate Composite. Chirality.

[57] Klingler R.J., Kochi J.K. (1981). Electron-Transfer Kinetics from Cyclic Voltammetry. Quantitative Description of Electrochemical Reversibility. J. Phys. Chem..

[58] Ibrahim N.I., Wasfi A.S. (2019). A Comparative Study of Polyaniline/MWCNT with Polyaniline/SWCNT Nanocomposite Films Synthesized by Microwave Plasma Polymerization. Synth. Met..

[59] Scandurra G., Antonella A., Ciofi C., Saitta G., Lanza M. (2014). Electrochemical Detection of P-Aminophenol by Flexible Devices Based on Multi-Wall Carbon Nanotubes Dispersed in Electrochemically Modified Nafion. Sensors.

[60] Ji D., Shi Z., Liu Z., Low S.S., Zhu J., Zhang T., Chen Z., Yu X., Lu Y., Lu D. (2020). Smartphone-Based Square Wave Voltammetry System with Screen-Printed Graphene Electrodes for Norepinephrine Detection. Smart Mater. Med..

[61] Guziejewski D. (2020). Electrode Mechanisms with Coupled Chemical Reaction—Amplitude Effect in Square-Wave Voltammetry. J. Electroanal. Chem..

[62] Üzer A., Sağlam Ş., Can Z., Erçağ E., Apak R. (2016). Electrochemical Determination of Food Preservative Nitrite with Gold Nanoparticles/p-Aminothiophenol-Modified Gold Electrode. Int. J. Mol. Sci..

[63] Yin H., Shang K., Meng X., Ai S. (2011). Voltammetric Sensing of Paracetamol, Dopamine and 4-Aminophenol at a Glassy Carbon Electrode Coated with Gold Nanoparticles and an Organophillic Layered Double Hydroxide. Microchim. Acta.

[64] Rattanarat P., Suea-Ngam A., Ruecha N., Siangproh W., Henry C.S., Srisa-Art M., Chailapakul O. (2016). Graphene-Polyaniline Modified Electrochemical Droplet-Based Microfluidic Sensor for High-Throughput Determination of 4-Aminophenol. Anal. Chim. Acta.

[65] Elancheziyan M., Senthilkumar S. (2021). Redox-Active Gold Nanoparticle-Encapsulated Poly(Amidoamine) Dendrimer for Electrochemical Sensing of 4-Aminophenol. J. Mol. Liq..

