# Supplementary material for: Development of an Efficient Voltammetric Sensor for the Monitoring of 4-Aminophenol Based on Flexible Laser Induced Graphene Electrodes Modified with MWCNT-PANI
Source: Sensors (Basel). 2022 Jan 22;22(3):833. doi: 10.3390/s22030833 (PMC8840637; doi:10.3390/s22030833)
Supplement: Supplementary file 1 [file sensors-22-00833-s001.zip › sensors-1491213-supplementary.pdf]

Development of an Efficient Voltammetric Sensor for the Monitoring of 4-Aminophenol based on Flexible Laser Induced Graphene Electrodes Modified with MWCNT-PANI

Salem Nasraoui [1, 2,*], Sami Ameur [2, 3], Ammar Al-Hamry [1], Mounir Ben Ali [2, 4], and Olfa Kanoun [1,*]

[1] Professorship Measurement and Sensor Technology, Chemnitz University of Technology, 09111 Chemnitz, Germany; ammar.al-hamry@etit.tu-chemnitz.de (A.A.-H.); olfa.kanoun@etit.tu-chemnitz.de (O.K.)
[2] University of Sousse, Tunisia and NANOMISENE Lab, LR16CRMN01, Centre for Research on Microelectronics and Nanotechnology of Sousse, Technopole of Sousse B.P. 334, Sahloul, Sousse 4034, Tunisia; mounir.benali@issatso.rnu.tn
[3] Higher Agronomic Institute of Chott-Mariem, University of Sousse, Sousse, Tunisia; sami.ameur@ISSACM.u-sousse.tn
[4] Higher Institute of Applied Sciences and Technology of Sousse, University of Sousse, 4003 Tunisia of Sousse, GREENS-ISSAT, Cité Ettafala, Ibn Khaldoun, Sousse 4003, Tunisia; mounir.benali@issatso.rnu.tn

* Correspondence: olfa.kanoun@etit.tu-chemnitz.de (O.K.); Tel.: +491795392601

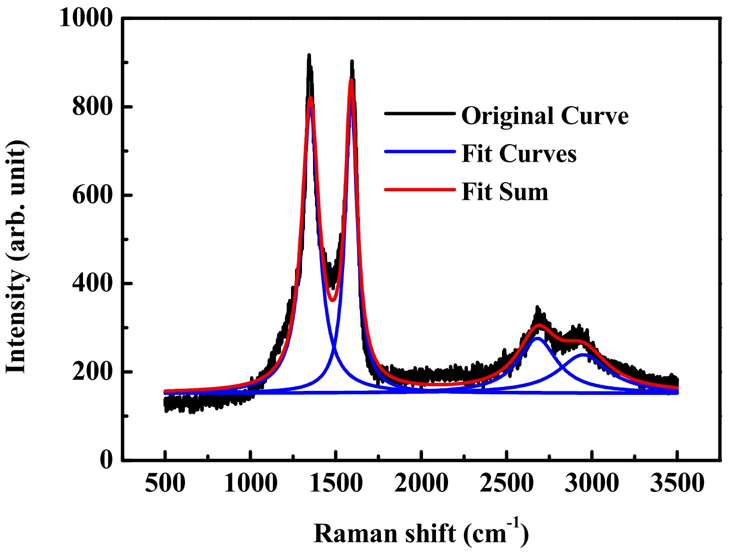

Figure S1. Raman spectrum of laser induced carbon.

The Raman spectroscopy was done to characterise the LIC electrode. The fitted spectra presents two Raman-region. The region one is below 2000 cm^{-1} related to D sp3 carbon atoms of disordered graphite and the G peak associated to the E2g mode of graphite referring to the in-plane vibration of sp2 carbon atoms in a 2D hexagonal lattice [1]. The second is linked to 2D and D+G peaks, the presence of defect-induced contributions is characterized by the 2D peak, whereas, the D+G combination peak strongly supports the presence of a higher disorder structure of graphene with oxygen-containing groups [2]. The atomic structure of laser induced carbon has been proposed to be a few-layers of graphene "pyrolytic carbon" [3] with a non-uniform scope of the oxygen-containing groups, coming about in sp2 carbon clusters of some nanometers disconnected inside an imperfect carbon grid or the sp3 network.

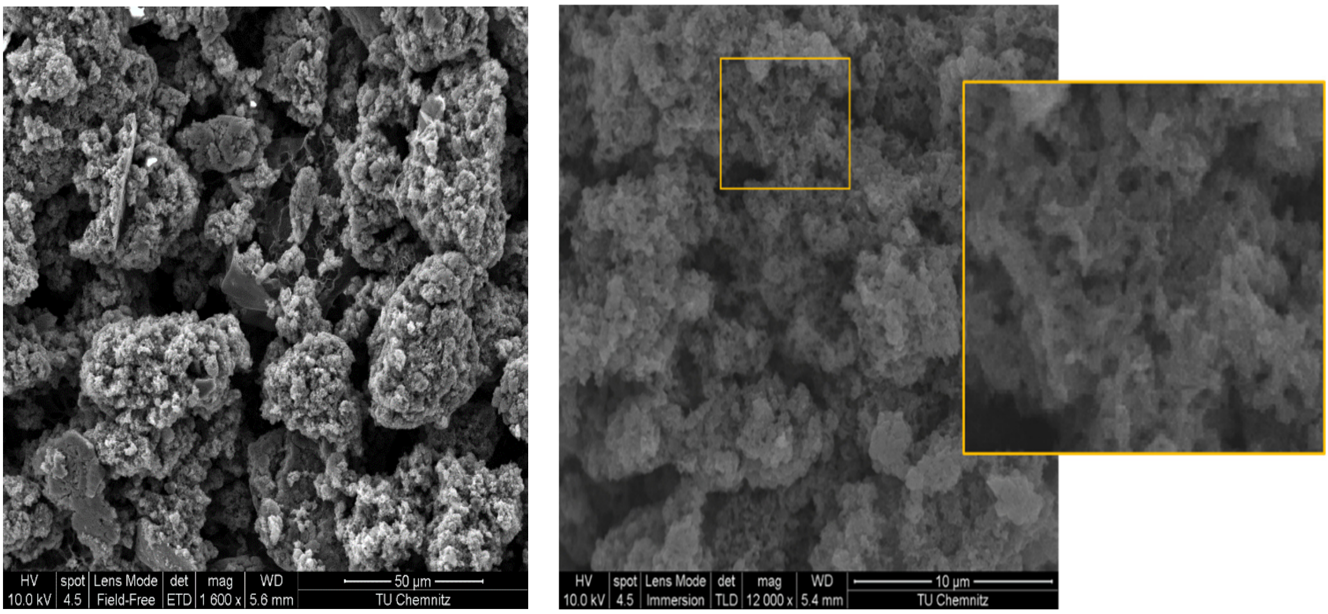

Figure S2. SEM of LIC/MWCNT-PANI.

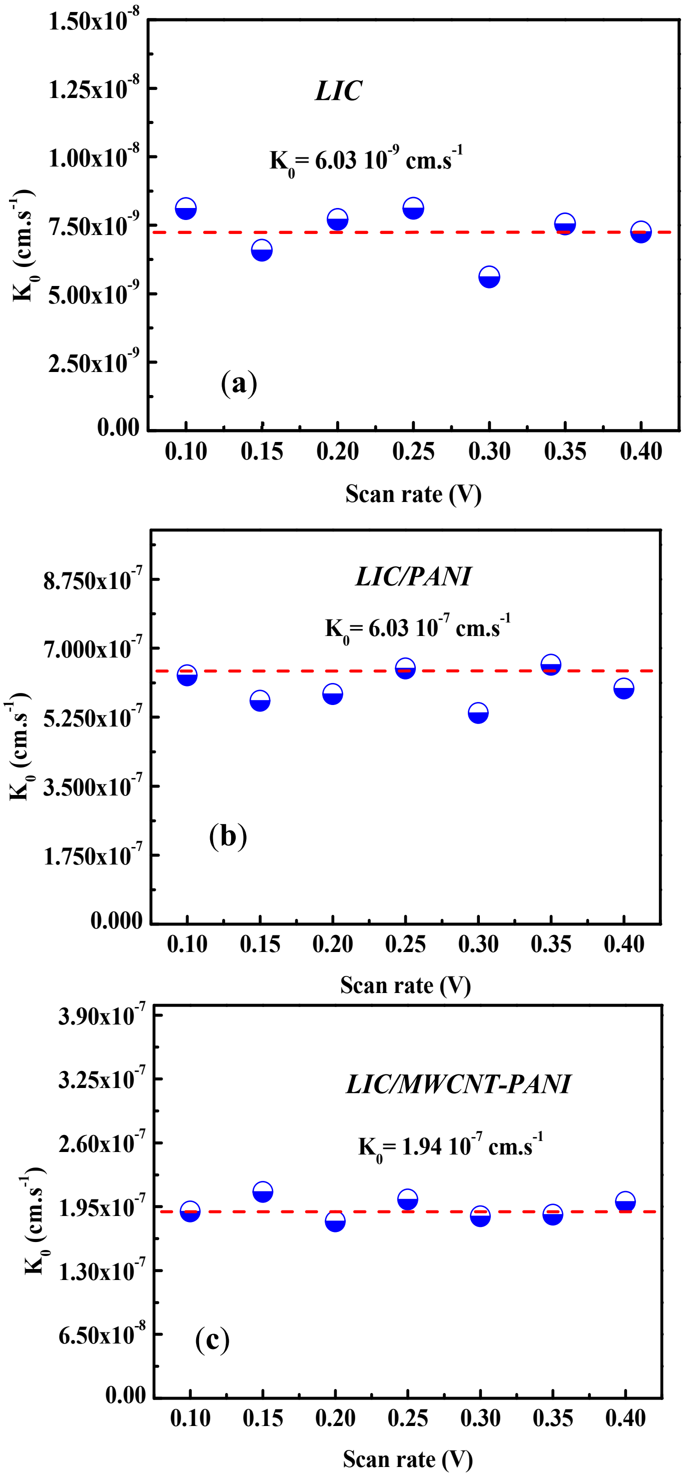

Figure S3. Klingler-Kochi analyses of the calculated ET rates (k0) for each scan rate.

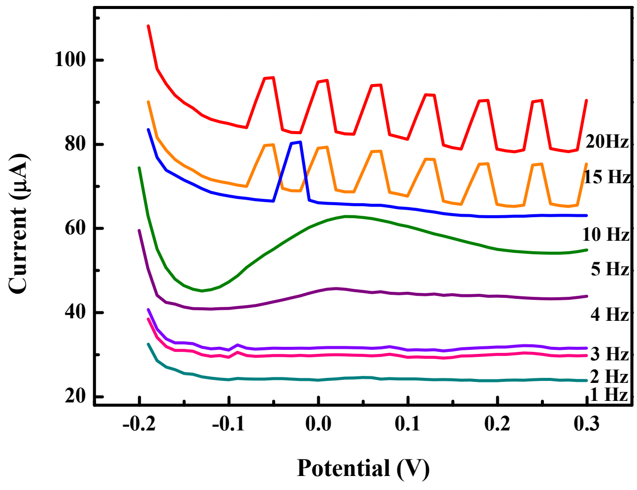

Figure S4. SWV of 1.0 μM 4-AP in PBS (pH=6.5) at different frequencies from 1.0 Hz to 20 Hz.

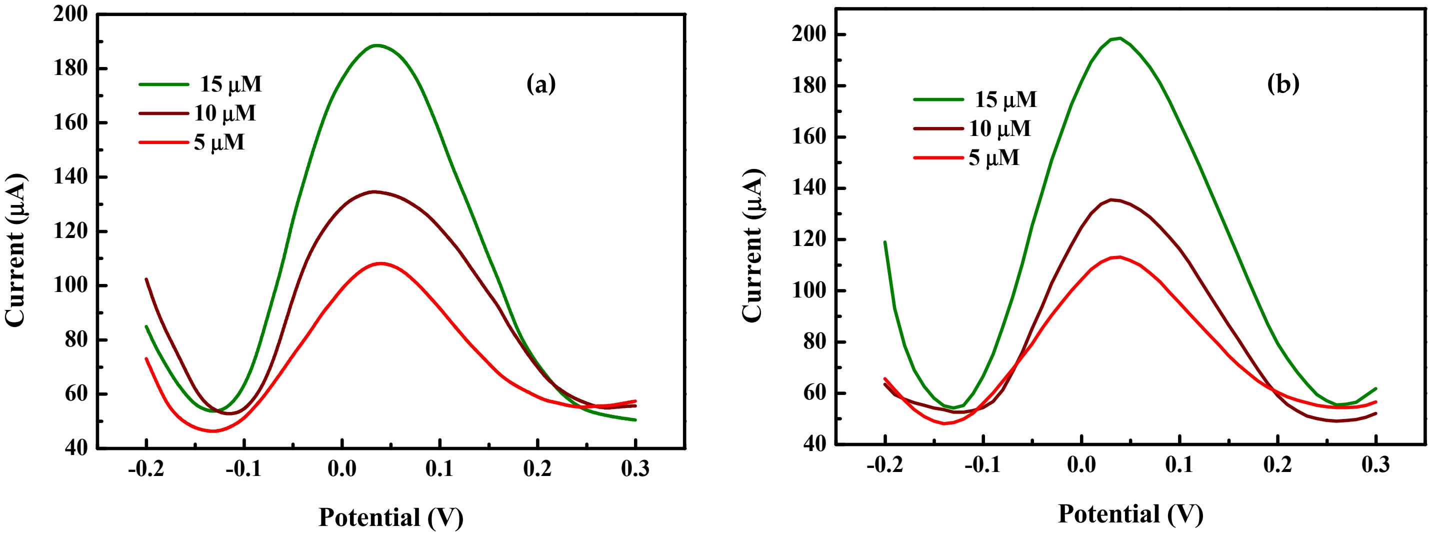

Figure S5. a) SWV in water samples **b)** in Paracetamol samples.

References

1. Albert, E.L.; Che Abdullah, C.A.; Shiroshaki, Y. Synthesis and Characterization of Graphene Oxide Functionalized with Magnetic Nanoparticle via Simple Emulsion Method. *Results in Physics* **2018**, *11*, 944–950, doi:10.1016/j.rinp.2018.10.054.
2. Bîru, E.I.; Iovu, H. Graphene Nanocomposites Studied by Raman Spectroscopy. In *Raman Spectroscopy*; Nascimento, G.M. do, Ed.; InTech, 2018 ISBN 978-1-78923-000-0.
3. Sahoo, S.K.; Mallik, A. Simple, Fast and Cost-Effective Electrochemical Synthesis of Few Layer Graphene Nanosheets. *NANO* **2015**, *10*, 1550019, doi:10.1142/S1793292015500198.
